# Is there an Exposure–Response Relationship for Nivolumab in Real-World NSCLC Patients?

**DOI:** 10.3390/cancers11111784

**Published:** 2019-11-13

**Authors:** Audrey Bellesoeur, Edouard Ollier, Marie Allard, Laure Hirsch, Pascaline Boudou-Rouquette, Jennifer Arrondeau, Audrey Thomas-Schoemann, Manuela Tiako, Nihel Khoudour, Jeanne Chapron, Frédérique Giraud, Marie Wislez, Diane Damotte, Audrey Lupo, Michel Vidal, Jérôme Alexandre, François Goldwasser, Michel Tod, Benoit Blanchet

**Affiliations:** 1 Department of Medical Oncology, Cochin Hospital, AP-HP, CARPEM, 75014 Paris, France; audreybellesoeur@gmail.com (A.B.); laure.hirsch@wanadoo.fr (L.H.); jerome.alexandre@aphp.fr (J.A.); francois.goldwasser@aphp.fr (F.G.); 2 Clinical Research Unity, Innovation, Pharmacology, Hôpital Nord, 42000 Saint-Etienne, France; edouard.ollier@univ-st-etienne.fr; 3 INSERM, U1059, Dysfonction Vasculaire et Hémostase, 42000 Saint-Etienne, France; 4 Department of Pharmacokinetics and Pharmacochemisty, Cochin Hospital, AP-HP, CARPEM, 75014 Paris, France; marie.allard@aphp.fr (M.A.); manuelatiako@gmail.com (M.T.); nihel.khoudour@aphp.fr (N.K.); michel.vidal@aphp.fr (M.V.); 5 Department of Clinical Pharmacy, Cochin Hospital, AP-HP, 75014 Paris, France; audrey.thomas@aphp.fr; 6 UMR8038 CNRS, U1268 INSERM, Faculty of Pharmacy, University Paris Descartes, PRES Sorbonne Paris Cité, 75006 Paris, France; 7 Department of Pneumology, Cochin Hospital, AP-HP, 75014 Paris, France; jeanne.chapron@aphp.fr (J.C.); marie.wislez@aphp.fr (M.W.); 8 Department of Pathology, Cochin Hospital, AP-HP, 75014 Paris, France; diane.damotte@aphp.fr (D.D.); audrey.lupo@aphp.fr (A.L.); 9 UMRS U1138, Centre de Recherche des Cordeliers, Université Paris Descartes, 75005 Paris, France; 10 Cochin Institute, INSERM U1016, 75014 Paris, France; 11 Department of Clinical Pharmacy, la Croix Rousse Hospital, Hospices civils de Lyon, 69002 Lyon, France; michel.tod@chu-lyon.fr; 12 Department of clinical Pharmacology, Claude Bernard Lyon 1 University, 69100 Villeurbane, France; 13 EMR 3738, Lyon-sud Medical School, Lyon 1 University, BP 12 Chemin du grand revoyet, 69921 Oullins, France

**Keywords:** lung cancer, nivolumab, pharmacokinetics, effectiveness, toxicity, PK/PD

## Abstract

Pharmacokinetic/pharmacodynamic data from real-world cohort are sparse in non small–cell lung cancer (NSCLC) patients treated with nivolumab. The aim of this prospective observational study was to explore the exposure-response relationship for effectiveness and toxicity of nivolumab in 81 outpatients with metastatic lung cancer. Nivolumab plasma trough concentrations (Cmin) were assayed at days 14, 28, and 42. Prognostic factors (including Cmin) regarding progression-free survival (PFS) and overall survival (OS) were explored using a multivariate Cox model. A Spearman’s rank test was used to investigate the relationship between Cmin and grade >2 immune-related adverse events (irAE). Mean nivolumab Cmin was 16.2 ± 6.0 µg/mL (*n* = 76), 25.6 ± 10.2 µg/mL (*n* = 64) and 33.4 ± 11.3 µg/mL (*n* = 53) at days 14, 28, and 42, respectively. No pharmacokinetic/pharmacodynamic (PK/PD) relationship was observed with either survival or onset of irAE. Multivariable Cox regression analysis identified Eastern Cooperative Oncology Group Performance Status (hazard ratio 1.85, 95%confidence interval 1.02–3.38, *p*-value = 0.043) and baseline use of corticosteroids (HR 8.08, 95%CI 1.78–36.62, *p*-value = 0.007) as independent risk factor for PFS and only baseline use of corticosteroids (HR 6.29, 95%CI 1.46–27.08, *p*-value = 0.013) for OS. No PK/PD relationship for nivolumab was observed in real-world NSCLC patients. This supports the recent use of flat dose regimens without plasma drug monitoring.

## 1. Introduction 

In the last few years, immunotherapy has provided a major treatment advance for lung cancer patients. In particular, inhibitors of programmed cell death 1 (PD-1) pathway have significantly changed treatment algorithms. Anti-PD-1 monoclonal antibodies bind to the PD-1 receptor and prevent interactions with its ligands PD-L1 and PD-L2, thereby releasing PD-1 pathway-mediated inhibition of the immune tumor response [1]. Nivolumab, a fully human immunoglobulin G4, can help to reinstate the antitumor immune response by targeting PD-1 receptors located on lymphocytes’ surfaces. Nivolumab is currently approved by the Food and Drug Administration for the treatment of advanced solid cancers such as melanoma, renal cell carcinoma, head and neck squamous cell carcinoma, urothelial cancer, colorectal cancer with microsatellite instability, and hepatocellular carcinoma [2]. Besides, two phase III trials showed improved overall survival (OS), response rate, and progression-free survival (PFS) with nivolumab (3 mg/kg, every two weeks) versus docetaxel in patients with advanced, previously treated squamous-cell and non-squamous non-small cell lung cancer (NSCLC) [3,4]. Nivolumab is currently approved as second-line treatment after chemotherapy in NSCLC patients.

Despite significant advances in immuno-oncology, there is still a long way to go for NSCLC treatment. Only 20% of NSCLC patients respond to nivolumab as a second line therapy, and approximately half of these responses are durable [3,4]. In a context of personalized medicine, many questions remain about the unmet need for predictors of response and toxicity with nivolumab. Even though PD-L1 expression is commonly used in daily clinical practice in immuno-oncology, it is not mandatory for nivolumab prescription. A reliable baseline biomarker is still lacking to provide binary discrimination of responsiveness to nivolumab treatment [5], which could prevent both toxicities in non-responders and unnecessary costs. Questions regarding an optimal nivolumab dosing regimen also warrant consideration in NSCLC patients. Nivolumab was first approved for a 3 mg/kg dosing regimen every two weeks (Q2W). The relatively flat exposure–response relationships of safety and efficacy over a large range of plasma exposure (for dose levels of 1–10 mg/kg Q2W) argue for a wide therapeutic margin of nivolumab in NSCLC patients [6]. A quantitative clinical pharmacology approach has provided evidence of a similar risk- benefit profile of a 240-mg flat dose Q2W and a 480-mg flat dose every four weeks (Q4W) relative to 3 mg/kg Q2W [7,8], which recently resulted in the approval of these new dosing regimens in NSCLC patients. However, this approach, based on pharmacokinetic/pharmacodynamic (PK/PD) simulation, was conducted from a database including patients enrolled in different clinical trials (phase I, II, and III studies). In contrast with pembrolizumab [9], no phase III study is currently published to confirm these new dosing regimens of nivolumab in NSCLC patients. In this context, there can be concern, in particular at extreme weights. A better understanding of PK/PD relationship of nivolumab in NSCLC patients from the “real-world” could help physicians to optimize plasma drug exposure and the risk/benefit ratio in a daily clinical practice. 

Regarding pharmacokinetic data and the exposure–response relationship, the main published data arise from clinical trials conducted with selected patients. In NSCLC nivolumab trials, Feng et al. identified no relation between the average concentration after the first injection (Cavg1) and survival [6]. They described a flat exposure-response relationship and a wide therapeutic index for doses ranging from 1 mg/kg to 10 mg/kg. Two clinical trials confirmed these results in metastatic melanoma patients treated with nivolumab [9,10]. On the contrary, Basak et al. [11] recently published a correlation between nivolumab exposure and effectiveness (response and survival) in NSCLC patients from daily clinical practice. Thus, PK/PD data for nivolumab are currently conflicting between clinical trials and “real world” cohort studies. 

The aims of this prospective observational study were to explore the exposure–response relationship for effectiveness and toxicity of nivolumab in NSCLC patients in daily clinical practice and to characterize the interindividual variability in nivolumab plasma exposure. 

## 2. Results 

### 2.1. Study Population

**Figure 1:**
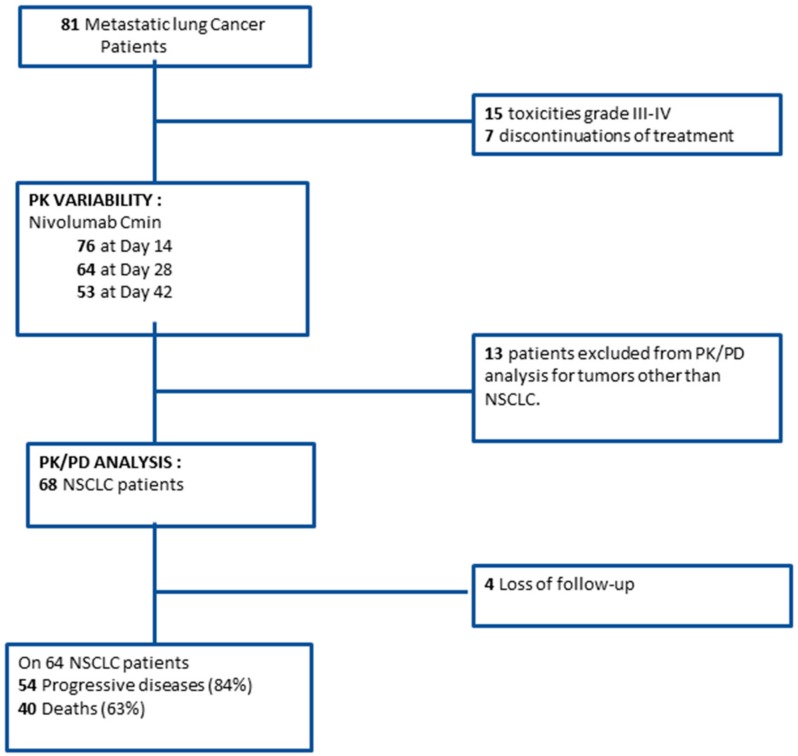
Study flowchart. PK/PD: pharmacokinetic/pharmacodynamic; NSCLC: non-small cell lung cancer.

Eighty-one patients with lung cancer were included in this study (Figure 1). 

The demographical, biological, and clinical characteristics of the cohort are summarized in Table 1. Forty-nine patients (60%) were male, with a median age of 65 years; 68 patients (84%) had NSCLC, and 20 patients (25%) experienced cerebral metastasis. Fifty-eight patients (72%) received nivolumab as second-line treatment. The results of PD-L1 testing were available for 43 patients (53%), with a median of 5% PD-L1 expression (0–28). 

Results are expressed as median (interquartile range) or frequency (percent).

In the whole population, the median duration of nivolumab treatment was 2.5 months (1.4–7.9). The median follow-up duration was 9.4 months (3.8—16.7). At data cut-off in January 2018, eight patients (10%) were still treated with nivolumab.

### 2.2. Pharmacokinetic Variability

**Figure 2:**
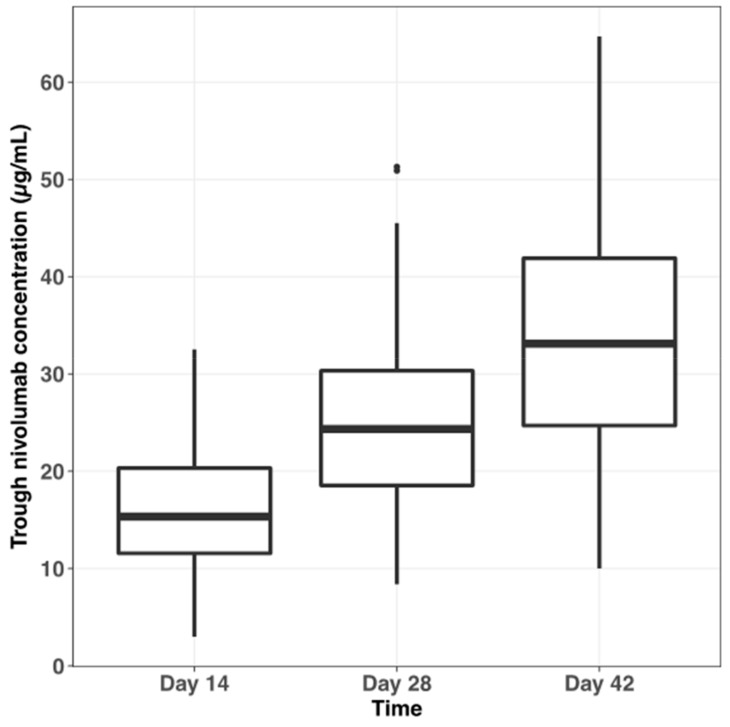
Plasma trough concentration of nivolumab in the whole cohort (*n* = 81 patients) within the first 42 days of treatment.

Pharmacokinetic data of the whole cohort (*n* = 81 patients) before the second (Day 14), third (Day 28), and fourth (Day 42) infusions of nivolumab after treatment initiation were analyzed to investigate the interindividual variability in trough plasma concentration (C_min_) of nivolumab and its determinants. Overall, 193 plasma samples could be analyzed (mean of 2.4 samples per patient). Figure S1 presents individual nivolumab plasma C_min_ within the 42 first days of treatment. Mean nivolumab plasma C_min_ at days 14, 28, and 42 after treatment start was 16.2 ± 6.0 µg/mL (*n* = 76, coefficient of variation; CV = 37.0%), 25.6 ± 10.2 µg/mL (*n* = 64; CV = 39.8%), and 33.4 ± 11.3 µg/mL (*n* = 53; CV = 33.8%), respectively (Figure 2). Nivolumab plasma C_min_ were statistically different (interaction test, *p*-value < 0.001) between different sampling occasions (i.e., days 14, 28, and 42). 

All pharmacokinetic data were initially analyzed using a population pharmacokinetic approach. Given that only nivolumab plasma C_min_ were available, the analysis was conducted by fitting a previously published population pharmacokinetic model to estimate individual parameters [12]. However, the shrinkage of the empirical bayesian estimates was large, precluding an analysis based on these estimates. In univariate analysis, gender (*p*-value = 0.078), total body weight (*p*-value = 0.005), body mass index (BMI) (*p*-value < 0.0001), C-reactive protein (CRP) level (*p*-value = 0.0089), cerebral metastasis (*p* = 0.013), and albumin level (*p*-value = 0.0068) were identified as factors influencing nivolumab plasma C_min_ at day 14 (Table 2). In multivariate analysis, only BMI was identified as an independent variability factor of nivolumab plasma C_min_ (*p*-value < 0.0001), with a strong positive correlation between these two parameters. Interestingly, baseline use of corticosteroids was statistically associated with nivolumab plasma C_min_ at day 28 (*p*-value = 0.0117). Thus, an increase of 8.7 μg/mL (CI95% 2.0; 15.4) for nivolumab plasma C_min_ is expected in patients treated with corticosteroids. By contrast, the relationship was not statistically significant at day 42 (*p*-value = 0.3). 

### 2.3. Exposure-Survival Relationship

**Figure 3:**
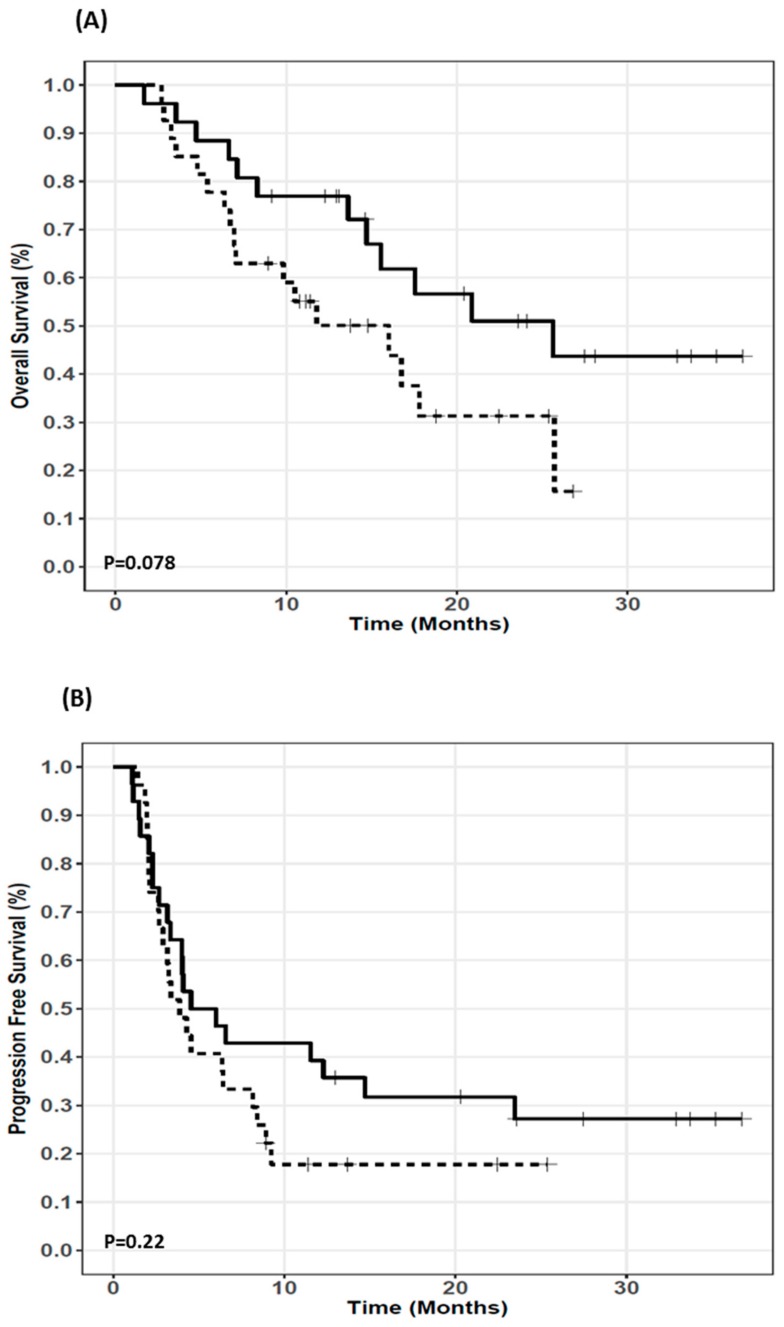
Kaplan-Meier curves of (**A**) overall survival and (**B**) progression-free survival according to plasma trough concentration of nivolumab at day 28 (≤24.7 µg/mL vs >24.7 µg/mL). The plain and dotted lines represent trough plasma concentration of nivolumab at day 28 ≤24.7 µg/mL (median value) and >24.7 µg/mL, respectively.

PK/PD data of patients with other histological tumor types than NSCLC (*n* = 13) were excluded for exposure-survival relationship analysis. Among NSCLC patients, four patients were lost to follow-up (Figure 1). Overall, the median PFS and OS in the PK/PD analysis cohort (*n* = 68 patients) were 4.0 months (CI95%: 3.2–6.4) and 15.5 months (CI95%: 8.3–20.9), respectively. Nivolumab plasma C_min_ (mean ± SD) at days 14, 28, and 42 after treatment start was 16.2 ± 6.0 µg/mL (*n* = 62, fifth to 95 th percentile: 6.5 to 25.1 µg/mL), 25.7 ± 10.2 µg/mL (*n* = 53; fifth to 95 th percentile: 10.5 to 42.6 µg/mL), and 33.4 ± 11.3 µg/mL (*n* = 43; fifth to 95th percentile: 16.4 to 50.9 µg/mL), respectively. NSCLC patients with C_min_ ≤ 24.7 µg/mL (median value) at day 28 tended to have longer OS (25.6 (15.5–undetermined) vs 16.0 (7.0-undetermined) months; *p*-value = 0.0786) (Figure 3A). By contrast, low nivolumab plasma C_min_ at day 28 (≤24.7 µg/mL) was not statistically associated with longer PFS (5.3 (3.3-undetermined) vs 3.9 (2.9–8.4) months, *p*-value = 0.22) (Figure 3B). There was no exposure-response relationship for C_min_ at day 14 or 42. 

**Figure 4:**
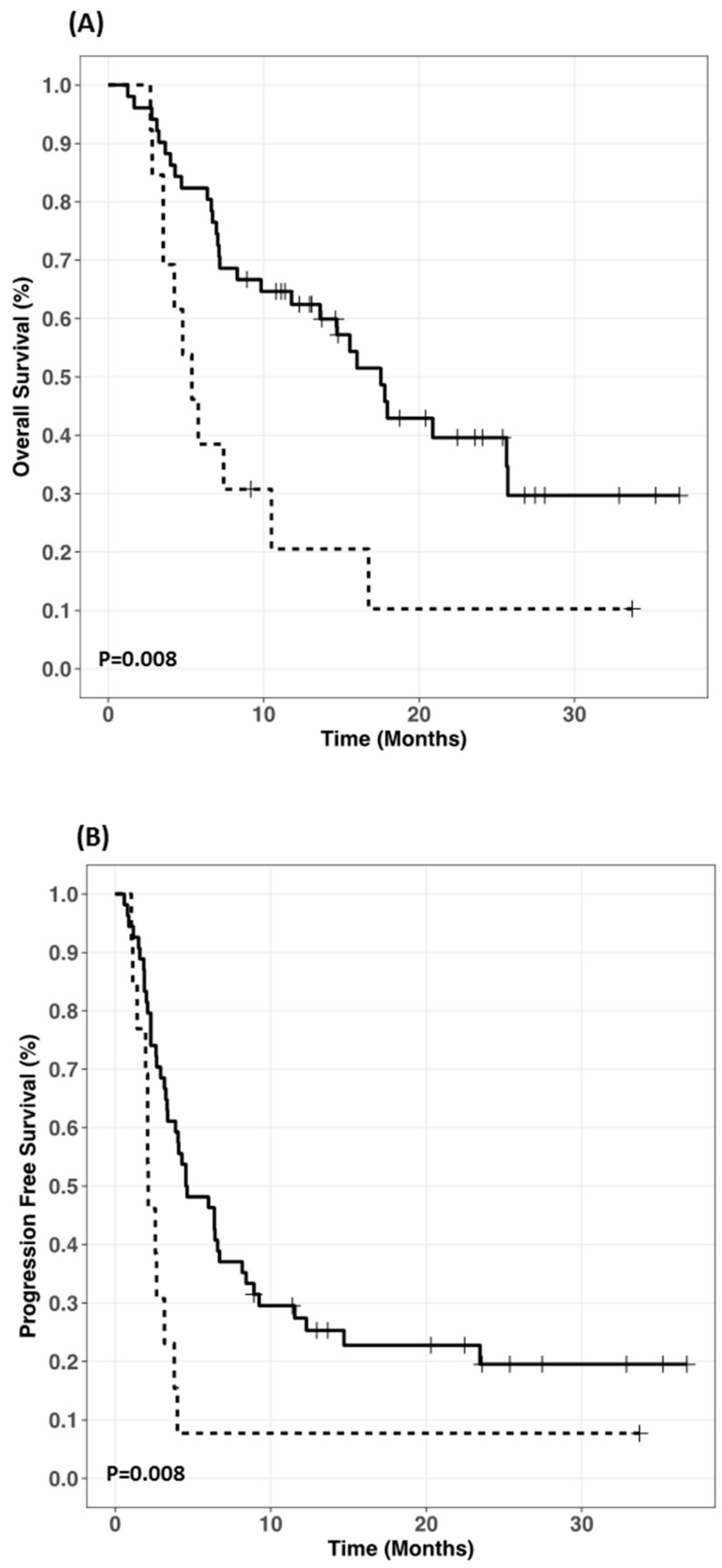
Kaplan-Meier curves of (**A**) overall survival and (**B**) progression-free survival according to systemic use of corticosteroids at initiation of nivolumab therapy. The dotted line represents non small–cell lung cancer patients concomitantly treated with corticosteroids.

Amongst 68 NSCLC patients, nine (13%) received systemic corticosteroids at the beginning of nivolumab therapy. Figure 4 shows that these patients had both shorter PFS and OS than corticosteroids free patients (PFS: 2.12 (1.96-undetermined) vs 4.58 (3.38–8.42) months; *p*-value = 0.008); (OS: 5.38 (3.54 undetermined) vs 17.54 (13.62 undetermined) months; *p*-value = 0.008). 

Multivariable Cox regression analysis identified Eastern Cooperative Oncology Group (ECOG) performance status (HR 1.85, 95%CI 1.02–3.38, *p*-value = 0.043) and baseline corticosteroids (HR 8.08, 95%CI 1.78–36.62, *p*-value = 0.007) as independent risk factor for PFS, and only baseline corticosteroids (HR 6.29, 95%CI 1.46–27.08, *p*-value = 0.013) for OS (Table 3). Finally, no PK/PD relationship was observed for survival in 59 free-corticosteroids NSCLC patients. The univariate analysis did not show any relationship between nivolumab plasma C_min_ at day 28 (≤24.7 µg/mL) and PFS (HR: 1.08 (0.54–2.16), *p*-value = 0.81) or OS (HR: 0.81 (0.34–1.90), *p*-value = 0.62). 

### 2.4. Exposure-Toxicity Relationship

Of 81 lung cancer patients, nivolumab treatment was associated in 15 patients (18%) with grade III or IV immune-related adverse events (irAE): cardiopulmonary events (5/81, 6%, with myocarditis, pericarditis, pulmonary hypertension, interstitial lung disease), colitis (3/81, 4%), rheumatologic disorders (2/81, 2%, arthritis), neurological events (2/81, 2%, peripheral neuropathy and central nervous system disorder), endocrine disorders (2/81, 2%, hypophysitis and diabetes), and renal insufficiency (1/81, 1%). Nivolumab was discontinued in seven patients—two patients at day 14 with respective C_min_ of 8.8 and 15.4 µg/mL, three patients at day 42 (C_min_ of 42.1, 32.1, and 38.3 µg/mL at the time of treatment discontinuation), and the two others after more than six months of treatment. For five patients with plasma C_min_ at the time of treatment discontinuation, C_min_ was included in the 10-90th percentile range of C_min_ observed in the whole cohort. Overall, no relationship was found between nivolumab plasma C_min_ and the occurrence of grade III or IV irAE.

## 3. Discussion 

Nivolumab treatment can enable durable responses and long-term overall survival in NSCLC patients [13]. However, predicting response to nivolumab remains currently difficult since some patients without or with low PDL-1 expression do have responses [5,14]. In this context, the identification of factors contributing to the interindividual variability in clinical outcomes of nivolumab is mandatory, especially in real world setting. The present study shows that use of corticosteroids at initiation of nivolumab therapy is a deleterious factor for NSCLC patients receiving nivolumab, while nivolumab plasma exposure does not seem to have any influence on both effectiveness and safety.

PK/PD data about PD-1 inhibitors remain sparse [2], especially in unselected NSCLC patients. Bajaj et al demonstrated that nivolumab clearance decreases over time [12]. Furthermore, this significant decrease in nivolumab clearance over time was identified as a predictive factor of response in patients with advanced solid tumors [10,12]. Baseline clearance has also been identified as a predictive factor of survival for anti-PD-1 antibodies [9,15]. The higher plasma exposure at steady state in responders could be the result rather than the cause of better response to nivolumab therapy. Indeed, decreased cachexia and improvement in disease state characterizing a lower tumor burden, normalization of protein turnover rate, and decreased inflammation state might contribute to the decrease in nivolumab clearance over time. Simulation has showed that exposure–response analysis results based on nivolumab plasma exposure after a single infusion was more consistent with the true exposure–response than with the steady-state plasma exposure [6,12,16].

In the present study, the multivariable Cox regression analysis failed to show a relationship between survival and nivolumab plasma C_min_ at day 14 or 28. A recent study suggests that a threshold value of 10 µg/mL of nivolumab would allow for >90% of maximum achievable receptor occupancy (about 80% receptor occupancy) of circulating T lymphocytes T [17]. Another study proposes a target concentration of 4.5 µg/mL [18]. In the present study, eight patients (11.2%) and none exhibited nivolumab plasma C_min_ below 10 µg/mL and 4.5 µg/mL at day 14, respectively. These results could explain in part the lack of relationship between nivolumab plasma C_min_ and survival in our cohort. Nevertheless, one cannot exclude that this relationship is different between pharmacodynamic activity on infiltrated T lymphocytes and nivolumab concentration in the tumor microenvironment. Further investigations are required to address this issue. In the present study, the occurrence of grade III or IV irAE was not statistically associated with increased nivolumab plasma C_min_, which suggests that the onset of severe irAE would be more related to intrinsic immune characteristics than plasma drug exposure. Our results from “real-life” NSCLC patients support the lack of PK/PD relationship for both efficacy and safety, previously described from nivolumab clinical trials in melanoma, lung cancer, or Hodgkin patients [6,7,9,10,19,20]. Recently, Basak et al. reported exposure relationship for response and survival but not with toxicity [11]. These results are in contradiction with ours about effectiveness. Even though our population seems close to that of Basak et al., no information about corticosteroids exposure is available on their population. Furthermore, their analysis of the exposure–response relationship does not account for clinical and biological factors in multivariate analysis, which could be a limiting factor in the interpretation of their results. Overall, our results are in favor of a wide therapeutic index and consistent with flat-dose regimens recently approved for nivolumab [2]. Given the wide therapeutic index of nivolumab [2], our results suggest that an approach of pharmacokinetically guided dose adjustment of nivolumab should not be necessary to improve efficacy in daily clinical practice. However, recent in silico data suggest that drug monitoring could help to postpone forthcoming courses of nivolumab by simulating the time by which the patient is likely to be underexposed with regard to target engagement [18]. Overall, the extension of the administration interval guided by drug monitoring could improve the safety of nivolumab without compromising its efficacy.

**Figure 5:**
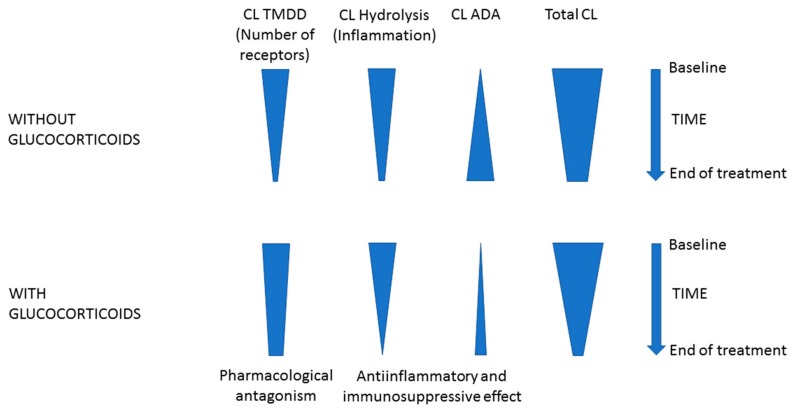
Factors influencing the time-course of nivolumab clearance during treatment. Total clearance is the sum of target-mediated clearance, hydrolysis clearance, and clearance due to antidrug antibodies. Each component varies with time, but the magnitude of the variation depends on the presence of glucocorticoids or not. See the discussion for further explanation. Abbreviations: ADA, antidrug antibody; CL, clearance; TMDD, target-mediated drug disposition.

The nivolumab exposure–response relationship may be confounded by several factors, including cachexia but also corticosteroids intake. The value of nivolumab clearance, which drives its plasma exposure, is the sum of the clearance in the tumor (target-mediated or proteolytic) [21], the hydrolytic clearance in other organs, and marginally, the clearance related to anti-drug antibodies (ADAs) [22] (Figure 5). Tumor-associated clearance is expected to be proportional to tumor size; hydrolytic clearance is known to decrease as inflammation regresses and ADA-mediated clearance increases over time if ADAs are produced [23]. Hypoalbuminemia is a well-recognized marker of cachexia and elevated protein turnover secondary to chronic systemic inflammatory conditions, as observed in many cancer indications. In contrast with other studies [12,24], we failed to identify albuminemia as an independent variability factor of nivolumab pharmacokinetics, probably because of the low interindividual variability in albuminemia. Depending on the time course of tumor size, inflammation status and ADAs production, nivolumab clearance may vary more or less. Moreover, the magnitude of the variation depends on the presence of glucocorticoids or not (Figure 5). On one hand, corticosteroids, by their anti-inflammatory and immunosuppressive properties [25], could decrease hydrolytic and ADA-mediated clearance. On the other hand, corticosteroids may antagonize the immunostimulant effect of nivolumab, resulting in a smaller antitumoral effect and a smaller reduction of the target-mediated clearance. In our data, corticosteroids tended to be associated with higher nivolumab plasma C_min_ at D14, D28, and D42 (significantly at D28). The lack of significant association between nivolumab plasma C_min_ at D42 and corticosteroids intake is unclear, it could be related to a lower statistical power because of many missing data. The statistical association between corticosteroids and higher nivolumab plasma C_min_ at D28 is consistent with nivolumab clearance being reduced by corticosteroids. However, the immunosuppressive effect of corticosteroids may also limit nivolumab pharmacodynamics, resulting in less tumor shrinkage and less tumor-associated clearance reduction. 

The second result of our study demonstrated that NSCLC patients treated with corticosteroids at the time of nivolumab initiation exhibited a shorter survival (PFS and OS) than in corticosteroid-free patients. Baseline use of corticosteroids, even at low dose (median dose in our population: 20 mg/d, range 10–25), was identified as an independent risk factor for PFS and OS in multivariate analysis, which confirms the results from recent studies [26,27]. Several hypotheses can be made to explain the detrimental effect of corticosteroid. First, baseline corticosteroid use could reflect a more severe condition associated with poorer prognosis independently of which anticancer drug is used. Thus, severe bone pain or intracranial hypertension are common indications of systemic corticosteroids. However, other parameters related to advanced disease, such as number of metastatic sites reflecting tumor burden, is associated neither with progression nor survival. 

Systemic corticoids could impair efficacy of PD1/PDL1 inhibitors by a pharmacokinetic or a pharmacodynamic interaction. In our study, corticosteroids use is not clearly associated with nivolumab exposure variability, which seems not to favor a pharmacokinetic interaction. The shorter survival in patients concomitantly treated with corticosteroids could be further related to PD interaction. As previously shown in transplant recipients, corticosteroids are able to decrease the immune reaction [25], which represents a pharmacodynamical antagonism for nivolumab. However, mechanisms of how corticosteroids prevent response to nivolumab remain ill-defined, and in vivo data are lacking. Different hypothesis among others can be proposed. Granzyme B (GranB) is a main component of the cytotoxic activity of CD8^+^ T cells and natural killer (NK) cells during the cellular immune response against cancer cells. Thus, recent studies have suggested potential benefit of GranB positron emission tomography (PET) imaging to predict response to cancer immunotherapy [28,29]. In renal transplant recipients treated with corticosteroids, Hruba et al. reported a significantly lower cytotoxic T lymphocytes and NK cells–derived GranB expression than in corticosteroid-free recipients at day 14 post-transplantation [30]. These data suggest that a lower baseline GranB expression is expected in NSCLC patients under corticosteroids at the time of nivolumab initiation, which could compromise the immunological effect of nivolumab. Costantini et al. recently showed an association between low plasma level of soluble GranB at nivolumab initiation and poor response, PFS and OS in 43 NSCLC patients treated with nivolumab [31], which supports our hypothesis. Finally, corticosteroids use is known to significantly decrease NK cells and/or impair their function in transplant recipients [30,32]. A high baseline level of circulating NK cells has been recently identified as a predictive factor of prolonged survival outcome in 31 NSCLC patients treated with nivolumab [33]. In the present study, we did not find any difference in baseline levels of circulating NK cells between NSCLC patients treated or not with corticosteroids (data not shown). Further investigations are warranted to elucidate how corticosteroids prevent nivolumab efficacy. However, our results suggest prudent use of corticosteroids at the time of nivolumab initiation. 

## 4. Materials and Methods

### 4.1. Participants

From July 2015 to June 2017, 81 outpatients treated for metastatic lung cancer in the CERTIM cohort (Immunomodulatory Therapies Multidisciplinary Study group) were included in this prospective observational monocentric study. All patients were treated and had follow-up visits in the oncology department of Cochin Hospital (Paris, France). Patients were eligible if they had a histologically confirmed lung cancer classified as stage IV or stage III without any curative treatment possible, progressive after at least one line of chemotherapy. A minimal age of 18-years-old, ECOG performance status ≤2, and adequate renal and hepatic functions according to manufacturer recommendations were also required. Patients with small-cell lung cancer who underwent nivolumab therapy as part of a clinical trial were eligible for pharmacokinetic analysis and exposure–toxicity analysis only. This prospective project was in compliance with the Declaration of Helsinki and approved by the local medical ethical board on 22 December 2016 with approval number CLEC N2442. All patients gave their written informed consent to participate in the study.

### 4.2. Treatment

All patients were treated with the recommended dose of nivolumab (3 mg/kg every two weeks) administered intravenously over 60 minutes. All adverse events were graded using the National Cancer Institute Common Toxicity Criteria, version 4.0. Nivolumab treatment was continued until disease progression (either based on clinical or radiological criteria) or unacceptable toxicity. However, treatment discontinuation was authorized in the cases of complete response; the timing of such interruption was left to the discretion of physician.

### 4.3. Pharmacokinetic Measurements 

Nivolumab C_min_ in plasma was determined just prior to next infusion at Day 14, Day 28 and Day 42. The plasma was separated by centrifugation (4000 rpm, 10 min, 4 °C) within two hours after blood collection and then stored at −80 °C until the analysis. Nivolumab plasma C_min_ were assayed using a validated home-made enzyme-linked immunosorbent assay (ELISA) [34]. The calibration range for nivolumab assay was 5–100 μg/mL with a limit of detection at 3 μg/mL. The within- and between-day accuracy for internal quality controls (5, 20 and 75 μg/mL) was included between –7.5% and 3.5%; within- and between-day imprecision were less than 5% and 12% for three levels of internal quality control, respectively. Nivolumab concentration is stable over a three-month period when plasma is stored at −80 °C [34]. All plasma samples were analyzed within the month after blood collection. 

### 4.4. PD-L1 Expression Analysis

Tumor PD-L1 protein expression was evaluated retrospectively in pretreatment (archival or recent) tumor-biopsy or surgical resection specimens with the use of an automated immunohistochemistry assay that used rabbit monoclonal antihuman PD-L1 antibody (E1L3N Cell Signaling Technology—automat Leica; Leica Biosystems, Nanterre, France). Tumors were defined as PD-L1 positive when staining of the tumor-cell membrane (at any intensity) was observed at pre-specified expression levels of 5% or higher in a section that included at least 100 tumor cells for evaluation. As PD-L1 status is not mandatory for nivolumab prescription, results were not available for all patients. 

### 4.5. Study Endpoints 

The main objective was to explore the relationship between nivolumab plasma exposure and survival in NSCLC patients. The primary endpoint was OS, defined as the time from the first infusion of nivolumab to death from any cause. The secondary endpoint was PFS, defined as the time from nivolumab treatment initiation to documented progression event (either clinical or radiological progression) or death from any cause. Radiographic evidence of progression was defined according to modified Response Evaluation Criteria in Solid Tumors [35]. The secondary objectives were to investigate the exposure-response relationship for grade III or IV irAE and to characterize the magnitude of interindividual variability in nivolumab pharmacokinetics in “real-world” lung cancer patients.

### 4.6. Statistical Analysis

Descriptive statistics used median (interquartile range) for quantitative variables and percentages for qualitative ones. Patients with small-cell lung cancer were included in the pharmacokinetic analysis and exposure-toxicity analysis but were excluded from the exposure–survival analysis (Figure 1). The relationships between the nivolumab C_min_ at day 14 and different variables were tested using two sample Wilcoxon tests or Kruskal-Wallis tests for qualitative variables with two modalities or more than two modalities, respectively, and by Spearman rank correlation coefficient tests for quantitative ones. The following variables were tested: age, age >70 years old, gender, total body weight, body mass index (BMI), lean body mass [36], C-reactive protein (CRP), albumin, immunoglobulin G, hepatic enzymes (AST and ALT), and baseline use of corticosteroids (i.e., patients who received systemic corticosteroids at the time of nivolumab initiation), PD-L1 expression, cerebral metastasis, tumor histology (adenocarcinoma vs squamous cell carcinoma), stage disease (III vs IV). A stepwise multiple linear regression was then performed with variables significant with *p* = 0.10 in the first step included in the regression and variables significant with *p* = 0.05 kept in the final model. Both OS and PFS functions were estimated using the Kaplan-Meier method. Survival data were analyzed using a Cox proportional hazard regression model adjusted for established clinical and biological risk factors. Following biological and clinical parameters were studied as prognostic factors: nivolumab plasma C_min_ at day 14 (<median value), nivolumab plasma C_min_ at day 28 (<median value), ECOG performance status [37,38], number of metastasis [38,39], neutrophil-to-lymphocyte ratio (NLR) (<median value) [39,40], and baseline use of corticosteroids and tumor PD-L1 protein expression (>median value) [14,41]. A Spearman’s rank test was used to investigate the relationship between nivolumab plasma C_min_ and grade >2 irAE. All computations were performed using R statistical software v3.4.4 (R Stats Package, R Foundation, Vienna, Austria).

## 5. Conclusions

Our results show a lack of PK/PD relationship, suggesting a safe use of flat-dose regimens. The wide therapeutic index of nivolumab supports a close benefit-safety profile between new dosing regimens (240-mg flat dose Q2W and 480-mg flat dose Q4W) and 3 mg/kg Q2W in unselected NSCLC outpatients. By contrast, corticosteroid use at the time of nivolumab initiation, even in small amounts, was associated with a significantly shorter survival (OS, PFS). Thus, we argue for limiting corticosteroid use during nivolumab initiation. 

## Figures and Tables

**Table 1:** Demographic and baseline characteristics of patients.

**Characteristics**	***n* = 81**
**Demographic data**	
Sex, n (%)	
Male	49 (60)
Female	32 (40)
Age (years)	65.0 (57.0–69.0)
Body weight (kg)	69.0 (62.0–78.0)
BMI (kg/m^2^)	23.7 (21.7–26.5)
Lean body mass (kg)	51.7 (41.3–57.2)
ECOG performance status, n (%)	
0–1	45 (56)
2	36 (44)
Corticosteroids therapy at nivolumab initiation, n (%)	14 (17)
Corticosteroids daily dose (mg)	20 (10–25)
**Disease characteristics**	
Histological tumor type, n (%)	
Adenocarcinoma	52 (64)
Squamous cell carcinoma	16 (20)
Other	13 (16)
Number of previous treatment line, n (%)	
1	58 (72)
2	13 (16)
≥3	10 (12)
Metastasis, n (%)	
Synchronous	55 (68)
Metachronous	26 (32)
Number of extrathoracic metastatic sites, n (%)	
0	20 (25)
1	29 (36)
2	17 (21)
≥3	15 (18)
Cerebral metastasis, n (%)	
Yes	20 (25)
No	61 (75)
Tumor cells PD-L1 expression (%)	5 (0–28)
**Baseline Biological data**	
Hemoglobin (g/dL) (*n* = 80)	12.2 (11.2–13.3)
Platelets (×10^9^/L) (*n* = 80)	244 (199–322)
Lymphocytes (×10^9^/L) (*n* = 74)	1.18 (0.85–1.72)
Neutrophils (×10^9^/L) (*n* = 80)	5.31 (3.89–6.84)
NLR (*n* = 74)	4.31 (2.99–5.67)
IgG (UI/mL) (*n* = 77)	10.2 (7.6–13.2)
PAL (UI/L) (*n* = 78)	87 (71–109)
AST (UI/L) (*n* = 78)	25 (21–31)
ALT (UI/L) (*n* = 78)	22 (18–35)
Total bilirubin (µmol/L) (*n* = 79)	5.9 (4.3–7.1)
Albumin (g/L) (*n* = 81)	38 (34–42)
CRP (mg/L) (*n* = 77)	11.2 (3.4–31.9)
Creatinine (µmol/L) (*n* = 81)	78 (64–90)

ALT, alanine amino transferase; AST, aspartate amino transferase; BMI, body mass index; CrCl, creatinine clearance; CRP, C-reactive protein, ECOG, Eastern Cooperative Oncology Group; IgG, immunoglobulin G; NLR, neutrophil to lymphocyte ratio; PD-L1, Programmed death-ligand 1; PAL, phosphatase alkaline.

**Table 2:** Determinants influencing nivolumab plasma trough concentration at day 14 after treatment initiation.

	Univariate		Multivariate
	Linear regression coefficient estimate (95% CI)	*p*-value	*p*-value
Age (year) (*n* = 75)	0.016 (−0.084; 0.117)	0.746	
Age > 70 years old (*n* = 75)	1.54 (−1.77; 4.85)	0.357	
Sex (male) (n=75)	−2.48 (−5.25; 0.28)	0.0777	0.36
Total body weight (kg) (*n* = 75)	0.146 (0.045; 0.247)	0.005	
BMI (kg.m^-2^) (*n* = 75)	0.78 (0.48; 1.09)	0.0000016	**<0.0001**
Lean body mass (kg) (*n* = 75)	0.012 (−0.13; 0.15)	0.862	
CRP (mg/L) (*n* = 72)	−0.054 (−0.094; −0.014)	0.0089	0.69
Albumin (g/L) (*n* = 75)	0.42 (0.12; 0.72)	0.0068	0.10
IgG (UI) (*n* = 71)	−0.277 (−0.661; 0.108)	0.156	
AST (UI) (*n* = 72)	−0.043 (−0.159; 0.074)	0.47	
ALT(UI) (*n* = 72)	0.044 (−0.050;0.138)	0.352	
Creatinine clearance (mL/min) ^a^	0.019 (−0.025; 0.064)	0.388	
PD-L1 expression	−0.0163 (−0.0643; 0.0318)	0.498	
Stage III vs stage IV (reference = stage III)	−1.571 (−4.882; 1.740)	0.348	
Adenocarcinoma vs SCC	−2.036 (−5.578; 1.506)	0.255	
Cerebral metastasis (reference = no)	−3.946 (0.873; 7.018)	0.013	0.145
Baseline use of corticosteroids	2.1 (−1.55; 5.76)	0.255	

ALT, alanine amino transferase; AST, aspartate amino transferase; BMI, body mass index; CRP, C-reactive protein; PD-L1, Programmed death-ligand 1; SCC, Squamous Cell Carcinoma. ^a^ Creatinine clearance was estimated using the Cockcroft-Gault equation. In multivariate analysis, bold value denotes statistical significance at the *p* < 0.05 level.

**Table 3:** Univariate and multivariate Cox proportional hazard analysis of risk factors for death and progression.

	Univariate Model HR (95%CI)	*p*-Value	Multivariate Model HR (95%CI) (*n* = 46)	*p*-value
**Risk of death**				
C_min_ D14 ≤median (*n* = 62)	1.03 (0.55–1.96)	0.9145		
C_min_ D28 ≤median (*n* = 53)	0.51 (0.24–1.09)	0.0786	1.37 (0.44-4.32)	0.586
ECOG Performance Status (*n* = 64)	2.06 (1.28–3.30)	0.0034	1.75 (1.86–3.57)	0.122
Number of metastasis (*n* = 64)	1.04 (0.80–1.35)	0.7987		
NLR > median (*n* = 58)	2.38 (1.05–5.55)	0.0036	1.82 (0.74–4.54)	0.189
PD-L1 > 5% (*n* = 37) ^a^	0.43 (0.18–1.02)	0.055		
Baseline use of corticosteroids	2.62 (1.29–5.31)	0.008	6.29 (1.46–27.08)	**0.013**
**Risk of progression**				
Cmin D14 ≤ median (*n* = 65)	1.14 (0.66–1.96)	0.646		
Cmin D28 ≤ median (*n* = 55)	0.68 (0.37–1.27)	0.223	1.30 (0.49–3.39)	0.59
ECOG Performance Status (*n* = 67)	1.97 (1.28–3.02)	0.023	1.85 (1.02–3.38)	**0.043**
Number of metastasis (*n* = 67)	1.09 (0.87–1.37)	0.452	1.14 (0.70–1.88)	0.58
NLR > median (*n* = 60)	1.82 (1.02–3.22)	0.042		
PD-L1 > 5% (*n* = 38) ^a^	0.44 (0.20–0.96)	0.041		
Baseline use of corticosteroids	2.51 (1.30–4.89)	0.008	8.08 [1.78–36.62)	**0.007**

95%CI, 95% confidence interval; C_min_, plasma trough concentration of nivolumab; D14, day 14 after treatment start; D28, day 28 after treatment start; HR, hazard ratio; ECOG, Eastern Cooperative Oncology Group; NLR, neutrophil to lymphocyte ratio; NT, not tested; PD-L1, Programmed death-ligand 1. ^a^ PD-L1 was not included in the multivariate analysis because of the high proportion of missing values. In multivariate analysis, bold values denote statistical significance at the *p* < 0.05 level.

## Supplementary Materials

The following are available online at https://www.mdpi.com/2072-6694/11/11/1784/s1, Figure S1: Individual plasma trough concentration of nivolumab (*n* = 81 patients) within the 42 first days of treatment.
