# Supplementary material for: Is there an Exposure–Response Relationship for Nivolumab in Real-World NSCLC Patients?
Source: Cancers (Basel). 2019 Nov 13;11(11):1784. doi: 10.3390/cancers11111784 (PMC6895963; doi:10.3390/cancers11111784)
Supplement: Supplementary file 1 [file cancers-11-01784-s001.pdf]

Supplementary Materials

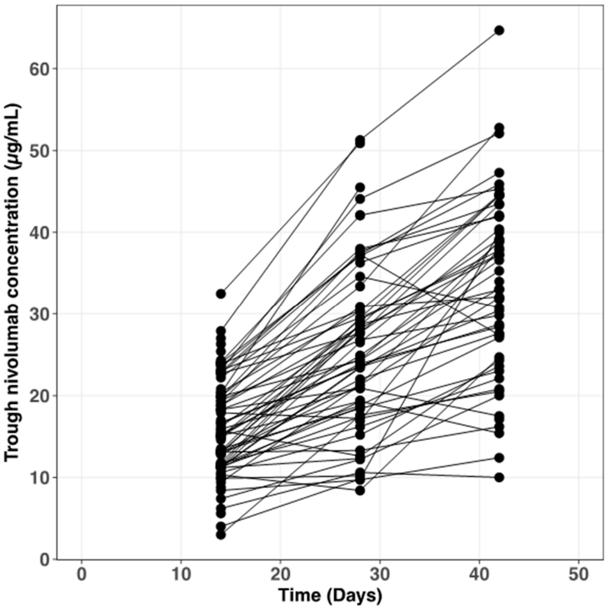

Figure S1. plasma trough concentration of nivolumab (*n* = 81 patients) within the 42 first days of treatment.
